# Using Open Dialogue-inspired dialogism in non-psychiatric medical practice: A ten-year experience

**DOI:** 10.3389/fpsyg.2022.950060

**Published:** 2022-08-24

**Authors:** Horacio J. Antoni

**Affiliations:** Federacion Agentina de Cardiologia, Tucumán, Argentina

**Keywords:** dialogism, Open Dialogue, anxiety, doctor-patient relationship, health psychology and medicine

## Introduction

Physicians are frequently consulted by people with physical symptoms that, after having ruled out an “organic” pathology, we suspect they are related to the most frequent psychological conditions in the usual consultation: the various forms of reaction to severe stress (Acute Stress Reaction and Adjustment Disorder, from ICD 11), “functional” pathologies, burn out syndrome, and anxiety disorders, especially Generalized Anxiety Disorder, with or without associated depression.

They are usually given a brief explanation about these problems and how they affect their health, given a brochure, or suggested a website with information. And then they are encouraged to visit a mental health practitioner. But there are some challenges to this seemingly simple scenario.

## The patients' confusion

Not all patients are ready or willing to hear that their health problem might be related to their life or emotions. Many are strongly influenced by mind-body dualism. Others have an intuition that such a relationship exists but bringing that to consciousness is not an easy process, because it can awaken emotions that are difficult to handle.

This is quite frequent in people suffering chronic anxiety (DSM-5 Generalized Anxiety Disorder) who prefer to consult medical providers rather than psychologists (Wittchen et al., 2002), because they do not consider their constant and excessive worry to be related to their discomfort. In their worrying they are hoping for a straightforward solution. Often these patients ask the physician for “solutions” to their symptoms in the form of medical treatments and they feel uncomfortable if one suggests there is a connection to their emotions.

## The doctors' confusion

We physicians know that listening to our stressed, distraught, or depressed patients is a noble and humanitarian task. But we are not convinced whether that listening has a real and proven effect on the patient's health. In a system that measures the “efficiency” of medical work by other parameters, and that increasingly takes more and more of our consultation time, listening tends to be overlooked. One usually asks general questions (verbally or through a questionnaire) about the level of stress, anxiety and depression, and then gives over-all information about these issues. But we don't know if one should ask about what is going on in their lives more specifically, moving from questioning to dialogue. Concretely, there are dilemmas that persist:

— Why, by what means does a conversation help the patient?
— Is this the competence of the physician or the psychologist?
— Is there a border or limit that should not be crossed?
— What should we “do” with all this “information” they are giving us?
— What does the patient really need from us when he/she tells us something personal?

To a non-physician reading this paper, these questions may seem weird. But physicians, in our medical training, have received different answers to these questions. Sometimes the teachers adhered to the theory that listening is something so complex, the human mind such an intricate and unconscious “mechanism of drives,” that it would be best not to enter such dangerous terrain and leave the task to the specialists. At the other extreme were those who proposed humanizing medicine, revaluing the doctor-patient relationship, empathizing with the patient… but these statements fell into vagueness and idealization, with no concrete way of putting them into practice, nor of verifying their efficacy.

## Patients in a spiral that progresses to grave consequences

Patients often come to the consultation in a strong emotional state where confusion, fear and discouragement predominate. They may not understand what is happening to them, and their thoughts are full of catastrophic anticipations. They suffer multiple discomforts due to the neurohumoral activation of stress: cardiovascular symptoms (hypertensive crises, tachycardia, shortness of breath, fainting), digestive symptoms (dyspepsia, gastritis, irritable bowel, etc.), dermatological and muscular among others.

They tend to dissociate physical symptoms from their emotional state, and this may generate a transitory benefit, but it ultimately increases their discomfort. They begin to believe that they have an uncertain and capricious pathology, which medicine can no longer decipher. If the physician restricts to prescribing drugs for each of these symptoms, a patient may leave the office with an endless list of medications that will have little effect. But most damagingly, it has reinforced the patient's belief that he/she is suffering from a “disease” in the most organic sense of the word.

If physicians do not have an appropriate conversation with these patients, it ends up creating a vicious circle: the worse the emotional state, the more physical symptoms are generated, creating a downward spiral. Often a depression secondary to stress appears, or preexistent conditions are exacerbated.

## A task that cannot be delegated

The psychologist is not responsible for providing medical information and clarity: it is up to us to explain the relationship between the autonomic activation of stress response and the emotional state. To confirm that the symptoms are an adaptive reaction of the body to stress, and not an “illness” on its own. In the case of these patients, the figure of the physician carries a lot of weight, a lot of power. I work together with psychologists and psychiatrists and many of my patients end up consulting them. But this first approximation is my responsibility. The physician is the bridge between the biological and the psychic world, the one responsible for breaking that harmful circularity. Nobody can do it for us if we don't. And doing so should not be optional, but part of the medical act, because the consequences can be serious.

## Discussion

It is also possible to conceptualize these patients' condition from a dialogic perspective (Hermans et al., 1992; Hermans, 2001; Seikkula, 2005; Antoni, 2022) where internal and external dialogicity are interrupted, and a monologic voice has taken control of their lives and suppressed other voices. For instance, a mother who suffers frequent severe hypertensive crises finds it difficult to relate this to the worry generated by an addicted child, or a violent intimate partner relationship. These are subjects triggering strong emotions and it is difficult to talk about them with others… but mainly with herself.

This woman's fear, anger, or exhaustion are present, but may be unlikely to surface in her consciousness, if the voice of the self-sacrificing mother or the devoted wife dominates the scene and becomes monologic. Denial or unawareness can function as a refuge from difficult-to-manage emotions, but this interrupted dialogicity occurs at the cost of great inner tension, that finally emerges as physical symptoms.

A dialogic way of listening stimulates the emergence of a polyphony of voices (Bakhtin, 2013) and emotions. External speech simultaneously activates inner one (Vygotsky, 1977; Riviere, 2005), the speech we use from childhood to order our conscience and regulate our actions. That allows “the speaker to inform his interlocutors on his experiences and at the same time shapes them and is more aware of them” (Seikkula, 2006, p. 102). In this way “speaking is an action in which the speaker allows himself to understand what he has said means to him” (p. 102). New meanings appear, alternative conceptualizations to the dominant narrative (Bruner, 1986, 1990; Charon, 2006).

The listener tries to respond to each word that is said, following the principle that the full sense of a sentence is reached with the response of the listener (Bakhtin, 2010; Seikkula and Arnkil, 2014). And as “symptoms inhabit emotions in the broad sense, in embodied emotions,” likewise “the new language arises also in experiences in the broad sense, in embodied experiences, and not in rational explanations” (Seikkula, 2006, p. 103).

## Concretizing dialogism in medical practice. First moment

Our first intention is also to listen to the patient in such a way that he/she begins to listen to him/herself, thus shaping his/her thoughts. Along with that, new voices and emotions appear, and more awareness and new meanings are generated.

In an initial moment or phase, where dialogic listening is paramount, I use resources such as reflections, affirmations, open questions and short summaries (Rogers, 2012; Miller, 2013). However, the most important resources are non-verbal: having eye contact, our body posture, and generating silences that lead the patient to think that what he or she is saying is clear, we understand and validate it.

I understand dialogicity as the situation that facilitates the emergence of the conditions of new insights, in a relational context. More than a means to produce new ideas, it is an integral human situation, where we intervene with all our corporeality and emotions. To dialogue is, above all, to help to create an emotional climate, where the patient feels confident to evoke difficult voices and see new alternatives. If this does not occur, everything remains on a rational level, as Seikkula says.

This requires the physician to participate in a less structured way than usual. Says Seikkula: “By responding as whole people, team members manifest that they are moved by the emotions in the room. Their calm and respectful conversational movements have a rhythm that allows them to fully experience and express the feelings in the meeting.” (2005, p. 466).

## Second moment

When patients have been able to speak, be heard and responded to; when they are more aware of the inner voices and the tension between them—the emotions involved-, then it is the physician's turn to give information. This is a second moment, so to speak. The doctor may explain the effects that emotions in general have on the activation of the neurohumoral stress response, and its expression in physical symptoms. The intention here is not to close or culminate the conversation, but to generate more dialogue based on this new information. I ask them: what do you think about what I have told you? Would you like to comment on it? And that always triggers new voices, more dialogue.

The physician's main objective for the patient is to find a relationship between these three factors: its own new polyphony of voices, the tension between them -the resulting emotions-, and the physical symptoms. This is usually experienced as a moment of enhanced awareness and clarity, even relief, because they can see a light of hope for their state of stagnation and pain. Stern (2004) terms this “present moment.” They calm them down, their fear of bodily symptoms diminishes, and even if they continue to suffer from them, they do not react with panic, they do not consider them a threat but part of a natural, adaptive response of their body to a stressful situation. The vicious circle (fear—more neurohumoral activation—more symptoms) is interrupted; and catastrophic anticipations and ruminations gradually diminish. Their own resources are activated, and they become more aware of the participation they may have in their own healing.

## Conclusions

Not all interviews are as linear as described, and each patient makes his/her own way as far as he/she is capable of. Even though, my 10-year experience of adapting Open Dialogue dialogism to my professional practice has confirmed that it is far superior to the psychodidactic one of cognitivism, which I have practiced before.

In the follow-up meeting most of the patients reported feeling better, calmer, and more hopeful. Also having been able to talk with their families and taking fewer medications for their problems. Some continue the conversation of the first consultation, and those who have finally consulted a psychologist have tripled in 5 years.

Regarding consultation time, it is difficult to dedicate 40–60 min to all patients. But just as we devote more time to severe and complex patients with organic pathologies than to others, we should consider and treat these patients in the same way. For other patients with a clearer awareness of the relationship with emotional states, a shorter conversation is sometimes sufficient.

I believe that we should not wait for health systems to take the first step in the direction of change, but be the ones to initiate that, even with a small number of patients to gain confidence in the model and experience.

I am convinced that Seikkula's vision of dialogism is a useful and feasible option to apply in our medical reality. And that it can make an enormous contribution to generate a more humane, more integral, and consequently, more efficient medicine.

## Author contributions

The author confirms being the sole contributor of this work and has approved it for publication.

## Conflict of interest

The author declares that the research was conducted in the absence of any commercial or financial relationships that could be construed as a potential conflict of interest.

## Publisher's note

All claims expressed in this article are solely those of the authors and do not necessarily represent those of their affiliated organizations, or those of the publisher, the editors and the reviewers. Any product that may be evaluated in this article, or claim that may be made by its manufacturer, is not guaranteed or endorsed by the publisher.
